# Impact of co-infection by hepatitis C virus on immunological and virological response to antiretroviral therapy in HIV-positive patients

**DOI:** 10.1097/MD.0000000000012238

**Published:** 2018-09-21

**Authors:** Julian Alexander Portocarrero Nuñez, Juan Gonzalez-Garcia, Juan Berenguer, María Jesús Vivancos Gallego, Jose Antonio Iribarren Loyarte, Luis Metola, Enrique Bernal, Gemma Navarro, Julia Del Amo, Inmaculada Jarrín

**Affiliations:** a Escuela Nacional de Sanidad, Instituto de Salud Carlos III; b Hospital Universitario La Paz/IdiPAZ; c Hospital Universitario Gregorio Marañón; d Hospital Ramón y Cajal, Madrid; e Hospital de Donostia, Donostia; f Hospital San Pedro, Logroño; g Hospital Reina Sofía, Murcia; h Corporació Sanitària Parc Taulí, Sabadell; i Centro Nacional de Epidemiología, Instituto de Salud Carlos III, Madrid, Spain.

**Keywords:** antiretroviral therapy, cohort study, Hepatitis C virus, HIV/AIDS, HIV/HCV co-infection, response to treatment

## Abstract

We assessed the effect of co-infection by hepatitis C virus (HCV) on immunological and virological response at 48 weeks from initiation of antiretroviral therapy (ART).

We included patients from the Cohort of Spanish HIV Research Network (CoRIS) starting ART between January 2004 and November 2014, had at least 1 CD4 T-cell count and viral load measurements both in the previous 6 months and at 48 (±12) weeks from ART initiation, and HCV serology before ART initiation. We used linear regression for mean differences in CD4 T-cell count increase from ART initiation and logistic regression to estimate odds ratios for virological response.

Of 12,239 patients by November 30, 2015, 5070 met inclusion criteria: 4382 (86.4%) HIV mono-infected and 688 (13.6%) HIV/HCV co-infected. Co-infected patients were more likely to have acquired HIV through injecting drugs use (57.4% vs. 1.1%), to be women, older, and Spanish, have a lower educational level, and having started ART with lower CD4 counts and acquired immunodeficiency syndrome. CD4 T-cell count increase at 48 weeks was 229.7 cell/μL in HIV-monoinfected and 161.9 cell/μL in HIV/HCV-coinfected patients. The percentages of patients achieving a virological response at 48 weeks were 87.0% and 78.3% in mono and coinfected patients, respectively. Multivariable analyses showed that at 48 weeks, coinfected patients increased 44.5 (95% confidence interval [CI]: 24.8–64.3) cells/μL less than monoinfected and had lower probability of virological response (odds ratio: 0.62; 95% CI: 0.44–0.88).

HIV/HCV-coinfected patients have lower immunological and virological responses at 48 weeks from ART initiation than monoinfected patients.

## 1 Introduction

Coinfection with hepatitis C virus (HCV) and human immunodeficiency virus (HIV) has been associated with a faster progression of hepatitis and higher liver-related mortality.^[1–3]^ However, the effect of co-infection on HIV progression and responses to HIV antiretroviral therapy (ART) seems less clear. Various studies have reported poorer immunological response to ART in HIV/HCV co-infected patients,^[4–11]^ whereas others have not found significant differences.^[12–15]^ Regarding the virological response to ART, most studies have not found significant differences, ^[7,8,12,16]^ although Hua et al^[9]^ reported a worse response in co-infected patients. It could be argued that inconsistent results might be explained by the heterogeneity of the populations in the different studies, the different study periods reflecting diverse ART regimens and small sample sizes.^[9,16]^ However, there are well-described biological mechanisms that could explain the different responses observed between coinfected and monoinfected individuals.^[1,17–19]^

The estimated global prevalence of HCV coinfection in HIV-positive individuals in 2013 was between 9.2% and 37.3%,^[20]^ and Spain was situated on the top of the rank in 2015.^[21]^ This very high prevalence of HCV coinfection in HIV-positive persons in Spain reflects a unique HIV epidemiological pattern pertaining the elevated numbers of persons who became infected through illicit drug use in the 1980s and 1990s which has declined over time, and with it, the proportion of co-infected subjects.^[22]^

Given the inconsistencies previously highlighted in the literature, the major changes in HIV clinical management in recent years and the lack of contemporary data addressing this question in Spain, we aimed to assess the effect of coinfection by HCV on immunological and virological response at 48 weeks from ART initiation in HIV-positive patients in the Cohort of the Spanish HIV Research Network (CoRIS) from January 2004 till November 2015. Additionally, we also modeled immunological responses in patients with optimal HIV responses to ART.

## 2 Methods

### 2.1 Study design, setting and participants

The Cohort of the Spanish HIV Research Network (CoRIS) is an open, multicenter, prospective cohort of HIV-infected individuals older than 13 years, naïve to ART at study entry, seen for the first time from 1 January 2004 at any of the 42 centers in 13 of the 17 autonomous regions in Spain. Subjects agree to participate in the study by signing an informed consent form. Ethics approval was obtained from all hospitals Ethic's Committees and every patient provides written informed consent to participate. CoRIS collects a minimum data set as described in the cohort protocol, which includes baseline and follow-up sociodemographic, immunological, and clinical data. Data are subjected to internal quality controls. Patients are followed periodically under routine clinical practice.^[23]^

**Figure 1:**
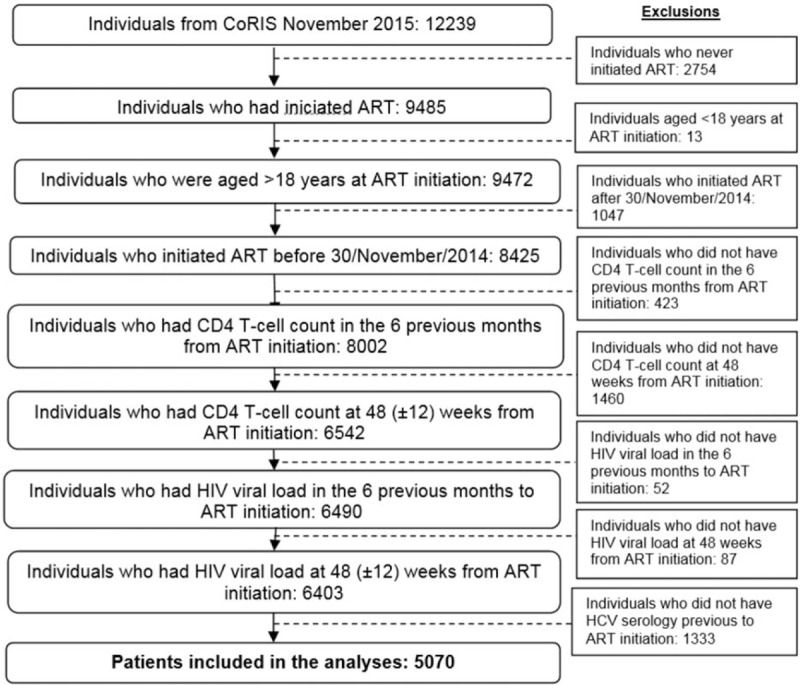
Flowchart of the study population.

Patients considered for inclusion were baseline ART naïve patients, aged ≥18 years, who started ART between 1 January 2004 and 30 November 2014 and had a CD4 T-cell count and an HIV viral load measurement both in the previous 6 months and at 48 (±12) weeks from ART initiation, and HCV serology previous to ART initiation. Because direct-acting antivirals (DAAs) were not widely available in Spain at the time of these analyses, none of the patients included had received them. Information regarding HCV treatment with interferon and ribavirin was not collected at the time and was, therefore, not taken into account in the analysis. A detailed flow chart is shown in Figure 1.

### 2.2 Variables and definitions

CoRIS collects information about birthdate, sex (male, female), geographical origin, education level (high school or lower, secondary, university, unknown), age, and year at start of ART, HIV-transmission category (men who have sex with men [MSM], injecting drugs users [IDU], heterosexual, unknown), CD4 T-cell count, HIV viral load, acquired immunodeficiency syndrome (AIDS) progression, hepatitis B surface antigen, hepatitis C antibodies, follow-up center, initial ART regimen, among others.

Subjects were classified at baseline as HIV-monoinfected and HIV/HCV-coinfected based on HCV serological findings.

### 2.3 Outcomes

Immunological response was defined as the change in CD4 T-cell counts at week 48 (±12) from ART initiation, and virological response as achieving a viral load ≤50 HIV-1 ribonucleic acid (RNA) copies/milliliter (mL) at 48 (±12) weeks from ART initiation.

### 2.4 Statistical analysis

A descriptive analysis of patients’ characteristics was carried out using frequency tables for categorical variables and median and interquartile range (IQR) for continuous variables. Differences in sociodemographic and clinical characteristics between HIV/HCV-coinfected and HIV-monoinfected patients were assessed through the nonparametric Mann–Whitney test for continuous variables and the *χ*^2^ test for independence for categorical variables.

Linear regression models were used to estimate the mean difference in the increase in CD4 T-cell counts at week 48 (± 12) from ART initiation, comparing HIV/HCV-coinfected versus HIV-monoinfected patients. Logistic regression models were used to estimate the odds ratio (OR) for the association between HIV/HCV coinfection and virological response at week 48 (±12) from the start of ART.

All the models were adjusted for potential confounders defined a priori: sex, age at start ART, HIV transmission category, geographical origin, educational level (a proxy of socio-economic status), CD4 T cells count pre-ART, HIV viral load pre-ART and AIDS pre-ART, hepatitis B infection pre-ART, and year at start ART. We assessed whether any of the variables studied modified the effect of HCV co-infection on immunological and virological response by including interaction terms in multivariable models.

We considered a possible interaction for period effect, in relation with 2 major changes on ART by 2008: extended use of single-tablet regimens (STR) that improves adherence and viral suppression^[24]^ and widespread introduction of integrase inhibitors.^[25]^ Because of that, we performed a sensitivity analyses, looking for the outcomes variables in both periods 2004 to 2007 and 2008 to 2015.

We considered the possibility of poor adherence in relation to no viral suppression, proposed in other studies, and modeled immunological responses in patients with optimal virological responses.

To adjust for clustering of patients within centres, robust methods were used to estimate standard errors and, thus, to calculate 95% confidence intervals (CI) and *P* values. Wald tests were used to derive *P* values. All statistical analyses were performed using Stata software (version 14.0; Stata Corporation, College Station, TX).

## 3 Results

### 3.1 Study population

CoRIS database, updated at 30 November 2015, included 12,239 individuals of whom 5070 met the inclusion criteria (Fig. 1), 4382 (86.4%) were HIV-monoinfected and 688 (13.6%) were HIV/HCV-coinfected patients. HIV/HCV-coinfected patients were more likely than those HIV-monoinfected to have acquired HIV through injecting drugs use, to be women, older at the start of ART, of Spanish origin with lower education level, and started ART with a lower CD4 count and an AIDS diagnosis (Table 1).

**Table 1:**
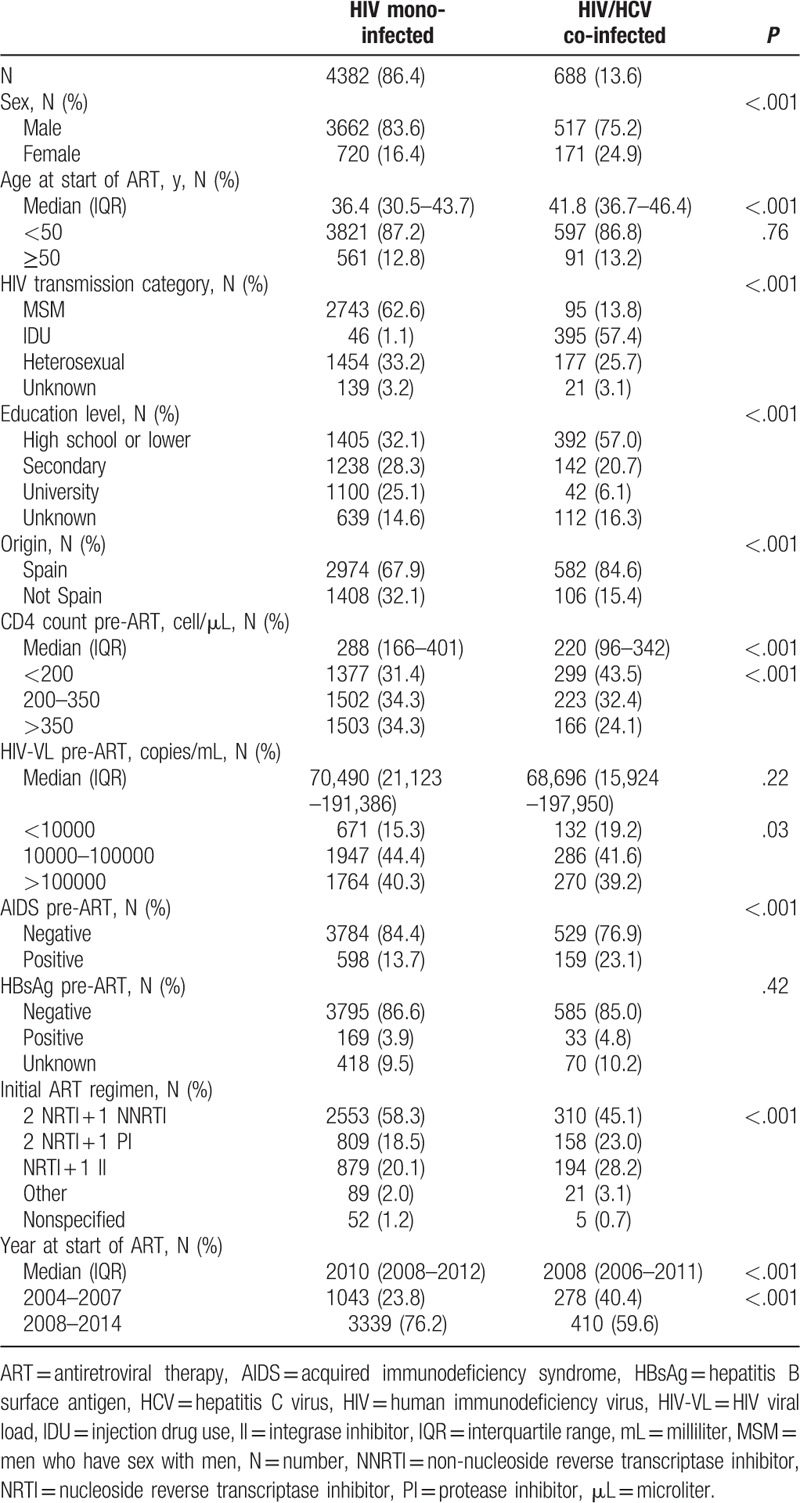
Sociodemographic and clinical characteristics.

### 3.2 Change in CD4 T-cell counts at week 48 from ART initiation

Mean (95% CI) CD4 T-cell increase at week 48 from ART initiation was 229.7 (224.2–235.2) cells/μL in HIV-monoinfected patients versus 161.9 (149.7–174.2) cells/ μL in HIV/HCV-coinfected patients (*P* < .001). In univariable analyses, the difference in mean CD4 T-cell count increase between HIV/HCV-coinfected and HIV-monoinfected patients was 67.8 (95% CI: 48.1–87.4) cells/μL. After adjustment for HIV transmission category, which appeared to be the only variable that confounded the association of interest, the difference in mean CD4 T-cell count increase was reduced to 44.5 cell//μL (95% CI: 24.8–64.3) but remained statistically significant (*P* < .001) (Table 2). We failed to find any evidence suggesting that any of the variables studied modified the effect of HCV-coinfection on the immunological response to ART, including a possible period effect (all interaction *P* values ≥.05).

**Table 2:**
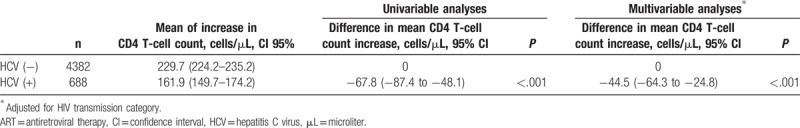
Impact of coinfection by HCV on immunological Response at 48 weeks from ART initiation.

We modeled immunological responses in patients with optimal virological responses, and we found that the difference in mean CD4 T-cell count increase was reduced to 37.1 cell//μL (95% CI: 15.1–59.2) but remained statistically significant (*P* = .002).

### 3.3 Virological response at 48 weeks after ART initiation

At 48 weeks from ART initiation, 87.0% of HIV-monoinfected patients and 78.3% of HIV/HCV-coinfected patients achieved virological response (*P* < .001). As for immunological response, HIV transmission category was the only variable that confounded the association of interest and after adjustment for this characteristic in a multivariable logistic regression model, HIV/HCV-coinfected patients still had a lower chance of achieving virological response than HIV-coinfected patients (adjusted OR 0.62; 95% CI 0.44–0.88; *P* < .01) (Table 3).

**Table 3:**
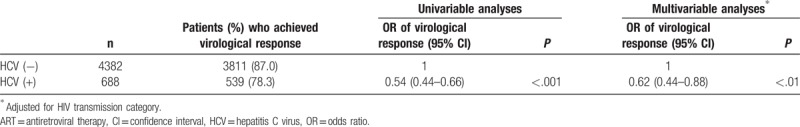
Impact of Co-infection by HCV on Virological Response at 48 weeks from ART initiation.

There was no evidence suggesting that any of the variables studied modified the effect of HCV-coinfection on immunological response, including a possible period effect (all interaction *P* values ≥.05).

## 4 Discussion

We found that HIV/HCV-coinfected patients within a national and representative cohort in Spain had poorer immunological and virological responses at 48 weeks from ART initiation than monoinfected patients. We observed these findings in crude and adjusted analyses, and saw no differential effect of HCV coinfection on HIV immunological and virological responses to ART from 2004 until 2015, before the widespread introduction of DAA's for the treatment of HCV infection in Spain.^[26]^ Consequently, we could not see its effects in this analysis.

Our results are consistent with the poorer immunological response at 48 weeks from ART initiation in HIV/HCV-coinfected compared to monoinfected patients reported in many studies, ^[7,8,10,11,16,27]^ although not in others.^[12]^ Of note, our results provide contemporary data supporting the inferior virological response in coinfected patients compared to monoinfected patients described by Hua et al^[9]^ 15 years ago. It must be mentioned, however, that most studies have not found statistically significant differences in virological responses to ART by HCV infection status^[7,8,10,12,16,27]^

Many possible reasons explaining the worse responses to ART of HIV/HCV-coinfected individuals have been proposed. Social factors, which differentially affect people dually infected with HIV and HCV, have been suggested as potential confounders or mediators. Additionally, biological factors connected to HCV pathogenicity are to be taken into account.

The social and behavioral factors associated with HCV infection, which could contribute to explain the worse responses to ART observed in our study include various forms of drug dependency,^[16,27]^ higher-risk sexual behavior,^[3]^ barriers to accessing health care,^[28]^ and poorer linkage and retention in care.^[29–33]^ In the present study, we have adjusted by HIV transmission category, the main behavioral factor, which accounted for the differences between groups, other social factors such as geographical origin and education level. Indeed, as far as we are aware, no previous study addressing this question has adjusted for this proxy of socioeconomic level. All other possible confounders have been evaluated like AIDS state, Hepatitis B co-infection, and the possibility of a period effect in relation to changes in ART (widespread introduction of STR and integrase inhibitors). Additionally, initial treatment regimen has been assessed, as it can affect the response (schedules that includes integrase inhibitors can achieve virological response faster than others), without finding significant differences between groups. Also, sensitivity analyses have been carried out, comparing immunological response between groups, only in the individuals with optimal virological responses, and the differences between groups remained. Also, Cescon et al^[34]^ compared the immunological and virological responses between HIV/HCV-coinfected IDU and HIV/HCV-coinfected non-IDU individuals, finding no differences in virological response and just a marginal difference in immunological response. This can support a direct biological effect of HCV on the responses.

Among the biological factors explaining the effect of HCV on ART responses, it has been found that in HIV/HCV co-infected patients, immune activation of memory CD4 T-cells is considerably increased,^[19]^ so is longer immune activation associated with high CD38 expression in T-cells, regardless of receiving ART.^[18]^ It has been described that HCV viral proteins can act as ligands for cell receptors of the immune system, among other cells, like HCV E2 protein can bind to the CD81 receptor and infect T-cells, B-cells, and monocytes.^[1,27]^ HCV core significantly seems to enhance HIV replication in human macrophages by upregulating Tumoral Necrosis Factor and interleukin-6.^[17]^ These mechanisms could explain the different evolution of the HIV infection in HIV/HCV-coinfected individuals.

As a limitation of the present study, information on HCV viral loads was not available for most patients, so we used HCV serology before ART initiation to classify them as mono or coinfected. In consequence, patients who spontaneously cleared HCV infection could have been wrongly classified as HIV/HCV coinfected patients. However, we do not think this might have biased our study as spontaneous clearance is believed to be around 10%—even lower among HIV/HCV co-infected patients^[35]^—and in any case, this misclassification bias goes toward the null hypothesis.

As strengths, this cohort includes patients from all over Spain with a broad spectrum of patient characteristics and antiretroviral regimens and is representative of the contemporary HIV epidemic. The overall number of patients was very high, limiting the random error found in other studies with smaller sample sizes. Similarly, this cohort was submitted to strict quality controls, limiting misclassification errors related to the collection of information. ART in the patients from the cohort is adjusted to national HIV treatment guidelines.^[23]^

The findings of this study have implications for clinical practice and public health. It is evident that HCV itself has a negative effect on the response to ART in HIV/HCV co-infected patients. Knowing that HCV infection has an effective treatment, boosting access to this treatment has an impact on HCV infection, but also has an impact on HIV infection and epidemic. As future work, repeating these analyses in the same cohort when HIV/HCV-coinfected patients have been treated for HCV may serve to strengthen the evidence of the need for HCV treatment, mainly as a public health approach in low-income countries where these 2 infections have a significant effect and/or where effective HCV treatment is not available.

## Acknowledgments

Centers and investigators involved in the Cohort of the Spanish HIV Research Network (CoRIS): Executive committee: Santiago Moreno, Julia del Amo, David Dalmau, Maria Luisa Navarro, Maria Isabel González, Jose Luis Blanco, Federico Garcia, Rafael Rubio, Jose Antonio Iribarren, Félix Gutiérrez, Francesc Vidal, Juan Berenguer, Juan González.

Fieldwork, data management and analysis: Paz Sobrino, Victoria Hernando, Belén Alejos, Débora Álvarez, Inma Jarrín, Nieves Sanz, Cristina Moreno.

BioBanK HIV: M Ángeles Muñoz-Fernández, Isabel García-Merino, Coral Gómez Rico, Jorge Gallego de la Fuente y Almudena García Torre.

Participating centres: Hospital General Universitario de Alicante (Alicante): Joaquín Portilla, Esperanza Merino, Sergio Reus, Vicente Boix, Livia Giner, Carmen Gadea, Irene Portilla, Maria Pampliega, Marcos Díez, Juan Carlos Rodríguez, Jose Sánchez-Payá.

Hospital Universitario de Canarias (San Cristobal de la Laguna): Juan Luis Gómez, Jehovana Hernández, María Remedios Alemán, María del Mar Alonso, María Inmaculada Hernández, Felicitas Díaz-Flores, Dácil García, Ricardo Pelazas., Ana López Lirola.

Hospital Universitario Central de Asturias (Oviedo): Victor Asensi, Eulalia Valle, José Antonio Cartón., Maria Eugenia Rivas Carmenado.

Hospital Universitario 12 de Octubre (Madrid): Rafael Rubio, Federico Pulido, Otilia Bisbal, Asunción Hernando, Maria Lagarde, Mariano Matarranz, Lourdes Dominguez, Laura Bermejo, Mireia Santacreu.

Hospital Universitario de Donostia (Donostia-San Sebastián): José Antonio Iribarren, Julio Arrizabalaga, María José Aramburu, Xabier Camino, Francisco Rodríguez-Arrondo, Miguel Ángel von Wichmann, Lidia Pascual Tomé, Miguel Ángel Goenaga, Mª Jesús Bustinduy, Harkaitz Azkune Galparsoro, Maialen Ibarguren, Maitane Umerez.

Hospital General Universitario De Elche (Elche): Félix Gutiérrez, Mar Masiá, Sergio Padilla, Andrés Navarro, Fernando Montolio, Catalina Robledano, Joan Gregori Colomé, Araceli Adsuar, Rafael Pascual, Marta Fernández, Elena García., Jose Alberto García, Xavier Barber.

Hospital Universitari Germans Trias i Pujol (Can Ruti) (Badalona): Roberto Muga, Jordi Tor, Arantza Sanvisens.

Hospital General Universitario Gregorio Marañón (Madrid): Juan Berenguer, Juan Carlos López Bernaldo de Quirós, Pilar Miralles, Isabel Gutiérrez, Margarita Ramírez, Belén Padilla, Paloma Gijón, Ana Carrero, Teresa Aldamiz-Echevarría, Francisco Tejerina, Francisco Jose Parras, Pascual Balsalobre, Cristina Diez.

Hospital Universitari de Tarragona Joan XXIII (Tarragona): Francesc Vidal, Joaquín Peraire, Consuelo Viladés, Sergio Veloso, Montserrat Vargas, Miguel López-Dupla, Montserrat Olona, Anna Rull, Esther Rodriguez-Gallego, Verónica Alba.

Hospital Universitario y Politécnico de La Fe (Valencia): Marta Montero Alonso, José López Aldeguer, Marino Blanes Juliá, Mariona Tasias Pitarch, Iván Castro Hernández, Eva Calabuig Muñoz, Sandra Cuéllar Tovar, Miguel Salavert Lletí, Juan Fernández Navarro.

Hospital Universitario La Paz-CarlosIII-Cantoblanco: Juan González-García, F Arnalich, José Ramón Arribas, José Ignacio Bernardino, Juan Miguel Castro, Luis Escosa, Pedro Herranz, Víctor Hontañón, Silvia García-Bujalance, Milagros García López-Hortelano, Alicia González-Baeza, María Luz Martín-Carbonero, Mario Mayoral, María José Mellado, Rafael Micán, Rocío Montejano, María Luisa Montes, Victoria Moreno, Ignacio Pérez-Valero, Berta Rodés, Talia Sainz, Elena Sendagorta, Natalia C Stella y Eulalia Valencia.

Hospital San Pedro Centro de Investigación Biomédica de La Rioja (CIBIR) (Logroño): José Ramón Blanco, José Antonio Oteo, Valvanera Ibarra, Luis Metola, Mercedes Sanz, Laura Pérez-Martínez.

Hospital Universitario Miguel Servet (Zaragoza): Ascensión Pascual, Carlos Ramos, Piedad Arazo, Desiré Gil.

Hospital Universitari MutuaTerrassa (Terrasa): David Dalmau, Angels Jaén, Montse Sanmartí, Mireia Cairó, Javier Martinez-Lacasa, Pablo Velli, Roser Font, Mariona Xercavins, Noemí Alonso.

Complejo Hospitalario de Navarra (Pamplona): María Rivero, Jesús Repáraz, María Gracia Ruiz de Alda, Carmen Irigoyen, María Jesús Arraiza.

Corporació Sanitària Parc Taulí (Sabadell): Ferrán Segura, María José Amengual, Gemma Navarro, Montserrat Sala, Manuel Cervantes, Valentín Pineda, Victor Segura, Marta Navarro, Esperanza Antón, Mª Merce Nogueras.

Hospital Universitario de La Princesa (Madrid): Ignacio de los Santos, Jesús Sanz Sanz, Ana Salas Aparicio, Cristina Sarriá Cepeda, Lucio Garcia-Fraile Fraile.

Hospital Universitario Ramón y Cajal (Madrid): Santiago Moreno, José Luis Casado, Fernando Dronda, Ana Moreno, María Jesús Pérez Elías, Cristina Gómez Ayerbe, Carolina Gutiérrez, Nadia Madrid, Santos del Campo Terrón, Paloma Martí, Uxua Ansa, Sergio Serrrano, Maria Jesús Vivancos.

Hospital General Universitario Reina Sofía (Murcia): Alfredo Cano, Enrique Bernal, Ángeles Muñoz.

Hospital Nuevo San Cecilio (Granada): Federico García, José Hernández, Alejandro Peña, Leopoldo Muñoz, Ana Belén Pérez, Marta Alvarez, Natalia Chueca, David Vinuesa, Jose Angel Fernández.

Centro Sanitario Sandoval (Madrid): Jorge Del Romero, Carmen Rodríguez, Teresa Puerta, Juan Carlos Carrió, Mar Vera, Juan Ballesteros.

Hospital Clínico Universitario de Santiago (Santiago de Compostela): Antonio Antela, Elena Losada.

Hospital Universitario Son Espases (Palma de Mallorca): Melchor Riera, Maria Peñaranda, Maria Leyes, Mª Angels Ribas, Antoni A Campins, Carmen Vidal, Francisco Fanjul, Javier Murillas, Francisco Homar.

Hospital Universitario Virgen de la Victoria (Málaga): Jesús Santos, Manuel Márquez, Isabel Viciana, Rosario Palacios, Isabel Pérez, Carmen Maria González.

Hospital Universitario Virgen del Rocío (Sevilla): Pompeyo Viciana, Nuria Espinosa, Luis Fernando López-Cortés.

Hospital Universitario de Bellvitge (Hospitalet de Llobregat): Daniel Podzamczer, Elena Ferrer, Arkaitz Imaz, Juan Tiraboschi, Ana Silva, Maria Saumoy.

Hospital Universitario Valle de Hebrón (Barcelona): Esteban Ribera.

Hospital Costa del Sol (Marbella): Julián Olalla, Alfonso del Arco, Javier de la torre, José Luis Prada, José María García de Lomas Guerrero.

Hospital General Universitario Santa Lucía (Cartagena): Onofre Juan Martínez, Francisco Jesús Vera, Lorena Martínez, Josefina García, Begoña Alcaraz, Amaya Jimeno.

Complejo Hospitalario Universitario a Coruña (Chuac) (A Coruña): Eva Poveda, Berta Pernas, Álvaro Mena, Marta Grandal, Ángeles Castro, José D. Pedreira.

Hospital Universitario Basurto (Bilbao): Josefa Muñoz, Miren Zuriñe Zubero, Josu Mirena Baraia-Etxaburu, Sofía Ibarra, Oscar Ferrero, Josefina López de Munain, Mª Mar Cámara. Iñigo López, Mireia de la Peña.

Hospital Universitario Virgen de la Arrixaca (El Palmar): Carlos Galera, Helena Albendin, Aurora Pérez, Asunción Iborra, Antonio Moreno, Maria Ángeles Campillo, Asunción Vidal.

Hospital de la Marina Baixa (La Vila Joiosa): Concha Amador, Francisco Pasquau, Javier Ena, Concha Benito, Vicenta Fenoll.

Hospital Universitario Infanta Sofia (San Sebastian de los Reyes): Inés Suárez-García, Eduardo Malmierca, Patricia González-Ruano, Dolores Martín Rodrigo.

Complejo Hospitalario de Jaén (Jaen): Mohamed Omar Mohamed-Balghata, Maria Amparo Gómez Vidal.

Hospital San Agustín (Avilés): Miguel Alberto de Zarraga.

Hospital Clínico San Carlos (Madrid): Vicente Estrada Pérez, Maria Jesus Téllez Molina, Jorge Vergas García, Elisa Pérez-Cecila Carrera.

Hospital Universitario Fundación Jiménez Díaz (Madrid): Miguel Górgolas., Alfonso Cabello., Beatriz Álvarez., Laura Prieto.

Hospital Universitario Príncipe de Asturias (Alcalá de Henares): Jose Sanz Moreno, Alberto Arranz Caso, Julio de Miguel Prieto, Esperanza Casas García.

Hospital Universitario Fundación Alcorcón (Alcorcón): Juan Emilio Losa, Maria Velasco Arribas, Leonor Moreno Núñez, Rafael Hervás Gómez.

Hospital Clínico Universitario de Valencia (València): Maria Jose Galindo Puerto, Ramón Fernando Vilalta, Ana Ferrer Ribera.

## Author contributions

**Conceptualization:** Julian Alexander Portocarrero Nuñez, Juan Gonzalez-Garcia, Juan Berenguer, Julia Del Amo, Inmaculada Jarrín.

**Data curation:** María Jesús Vivancos Gallego, Julia Del Amo, Inmaculada Jarrín.

**Formal analysis:** Julian Alexander Portocarrero Nuñez, Inmaculada Jarrín.

**Investigation:** Julian Alexander Portocarrero Nuñez, Juan Berenguer, María Jesús Vivancos Gallego, Jose Antonio Iribarren Loyarte, Luis Metola, Enrique Bernal, Gemma Navarro, Julia Del Amo, Inmaculada Jarrín.

**Methodology:** Julian Alexander Portocarrero Nuñez, Julia Del Amo, Inmaculada Jarrín.

**Project administration:** Julia Del Amo.

**Supervision:** Juan Gonzalez-Garcia, Juan Berenguer, Julia Del Amo, Inmaculada Jarrín.

**Writing – original draft:** Julian Alexander Portocarrero Nuñez, Julia Del Amo, Inmaculada Jarrín.

**Writing – review & editing:** Julian Alexander Portocarrero Nuñez, Juan Gonzalez-Garcia, Juan Berenguer, María Jesús Vivancos Gallego, Jose Antonio Iribarren Loyarte, Luis Metola, Enrique Bernal, Gemma Navarro, Julia Del Amo, Inmaculada Jarrín.
